# Notfälle in der Augenheilkunde: Vermittlung anhand interaktiver Key-feature-Fälle für Medizinstudierende

**DOI:** 10.1007/s00347-021-01409-1

**Published:** 2021-05-31

**Authors:** Andreas Müller, Felix M. Wagner, Alexander K. Schuster, Betül Günal, Norbert Pfeiffer, Franziska Schmidt, Verena Prokosch

**Affiliations:** 1 Augenklinik und Poliklinik der Universitätsmedizin Mainz, Johannes Gutenberg-Universität Mainz, Langenbeckstr. 1, 55131 Mainz, Deutschland; 2 Zentrum für Qualitätssicherung und -entwicklung, Johannes Gutenberg-Universität Mainz, Mainz, Deutschland; 3 Zentrum für Augenheilkunde, Uniklinik Köln, Köln, Deutschland

**Keywords:** Studium, Lehre, Prozedurales Denken, eLearning, Digital, Education, medical, Teaching, Procedural thinking, E‑learning, Digital

## Abstract

**Hintergrund:**

Wichtiges Ziel eines Curriculums für Medizinstudierende ist, die Fähigkeit zum selbstständigen Erkennen und Einordnen von Notfällen zu vermitteln. Die Augenheilkunde steht hierbei aufgrund fachspezifischer „red flags“, also Warnsymptomen und -zeichen, vor der Herausforderung, dass solche selten von anderen Organsystemen hierauf übertragen werden können. Um Medizinstudierende dabei zu fördern, die „red flags“ der Augenheilkunde in ihrer späteren Tätigkeit zu erkennen, entwickelten wir für unser eLearning-Angebot leitsymptomorientierte interaktive Fallvignetten.

**Material und Methoden:**

Es wurden 7 interaktive Fallvignetten zu potenziell bedrohlichen ophthalmologischen Symptomen und Zeichen wie „schmerzloser Visusverlust“ oder „rotes Auge“ entwickelt. Hierbei werden Studierende mit Bild und Text durch einen Fall geführt und zu entscheidenden Aspekten („key features“) mit verschiedenen Frageformaten geprüft. Die interaktiven Fälle wurden mithilfe von eLearning-Authoring-Software umgesetzt und als Lernmodule in der Learning-Management-Präsenz der Augenklinik integriert. Die Patientenfälle waren Teil unseres Praktikums der Augenheilkunde. Die Fälle wurden im Anschluss von den Studierenden evaluiert.

**Ergebnisse:**

Die Fälle wurden im Mittel mit einer Note von 1,51 ± 0,68 (Mittelwert ± Standardabweichung) bewertet (*n* = 163). Auf einer Likert-Skala wurden sie mit 1,60 ± 0,81 als hilfreich für das eigene Lernen empfunden (1 = sehr hilfreich, 7 = gar nicht hilfreich; *n* = 164). Die Informationsmenge und Auswahl der Szenarien wurden ebenfalls positiv evaluiert.

**Diskussion:**

Um Studierenden im engen zeitlichen Rahmen eines Kurses mehr Sicherheit im Erkennen und der primären Versorgung von augenärztlichen Notfällen verschaffen zu können, können praxisorientierte Key-feature-Fälle Bestandteil eines eLearning-Angebotes sein.

## Abstract (en)

**Background:**

Autonomous diagnosis and assessment of medical emergencies are important skills to acquire for medical students. Ophthalmology features certain specialty-specific “red flag” signs and symptoms, which pose a challenge for educators in ophthalmology. To support medical students in identifying those “red flags” we developed and implemented interactive cases for our e‑learning platform.

**Material and methods:**

A total of seven interactive cases with key feature problems regarding potentially dangerous signs and symptoms, such as painless loss of vision or red eye were developed. Medical students were guided through a case and performed formative assessments. The interactive cases were created with e‑learning authoring software and were available on the learning management system presence of the department of ophthalmology. They were mandatory for medical students in the ophthalmology course. Students evaluated the cases after the course.

**Results:**

The interactive cases were rated on average at 1.51 ± 0.68 (mean ± standard deviation; *n* = 163) on a grade scale (1 = best, 6 = worst). On a Likert scale they were perceived as helpful for individual learning at 1.60 ± 0.81 (1 = very helpful, 7 = not helpful at all; *n* = 164). The information provided on the cases and selection of scenarios was positively evaluated.

**Conclusion:**

To support students in identifying and managing ophthalmic emergencies in the context of limited time in tightly packed curricula, interactive key feature cases can be part of corresponding e‑learning resources. An integration of such cases was evaluated as desirable.

Das Erkennen und sachgerechte Einordnen von Notfällen ist zentrales Lernziel von medizinischen Curricula. Die Augenheilkunde hat hier eine Sonderstellung inne aufgrund der teils fachspezifischen Warnsymptome und -zeichen sowie Befundkonstellationen. Um Medizinstudierenden diese näher zu bringen, bearbeiteten die Studierenden im Sommersemester 2020 7 interaktive Patientenfälle und evaluierten diese.

Das Erkennen, korrekte Einschätzen und Management von medizinischen Notfällen ist ein grundlegendes Lernziel für Medizinstudierende und beliebter Gegenstand von Lehrforschung [4, 16, 17]. Da hierbei aufgrund des lebensbedrohenden Charakters häufig Erkrankungen wie Herzinfarkt oder polytraumatisierte Patienten im Vordergrund stehen, ist es notwendig, im Lehrkonzept der Augenheilkunde fachspezifische Notfälle und Warnsymptome symptom- und kompetenzorientiert im eigenen Curriculum zu verankern und zu vermitteln [14, 15]. Methodisch stehen hierfür verschiedene Lehrformate zur Verfügung, eine Möglichkeit ist die Verwendung von eLearning [1].

Um Studierende der Humanmedizin an der Universität Mainz darin zu unterstützen, ophthalmologische Notfälle und Warnsymptome, also „red flags“, besser zu erkennen und zu managen, entwickelten wir interaktive, leitsymptomorientierte Patientenfälle im Key-feature-Format. Im Key-feature-Format werden nach einer umfassenden Darstellung, z. B. der Vorgeschichte eines Patienten, immer wieder Fragen gestellt, welche kritische Abwägungen und Entscheidungen des Lernenden prüfen, die für die sachgerechte Versorgung eines Patienten notwendig sind. Klassische Fragen zu Key-Features beziehen sich auf Differenzialdiagnosen, diagnostische Untersuchungen, therapeutische Entscheidungen sowie auf das Management z. B. zeitkritischer Situationen [2, 6, 13].

Die interaktiven Fälle wurden in das humanmedizinische Curriculum für Augenheilkunde integriert. Im Sommersemester 2020 bestand ein Angebot von 7 interaktiven Patientenfällen, welches wir von den Studierenden evaluieren ließen.

## Methodik

Im Rahmen des Sommersemesters 2020 und der Corona-Pandemie wurde das Praktikum aufgrund der Einschränkungen des Präsenzunterrichtes vollständig digital durchgeführt. Die interaktiven Patientenfälle waren hierbei ein verpflichtender Bestandteil des Curriculums für das Praktikum der Augenheilkunde, welches an der Universitätsmedizin Mainz im sechsten Semester stattfindet. Da die interaktiven Fälle keinen Einfluss auf das Bestehen oder die Bewertung des Kurses haben, wurde kein spezifisches Standardlehrwerk zur Vorbereitung auf die Fälle angegeben. Mehrere ausgewählte Quellen (Lehrbuch, Amboss, Skript) wurden in unserer eLearning-Plattform angeboten, welche zur Vorbereitung oder Recherche genutzt werden konnten [11].

Weitere Bestandteile des Praktikums waren ein wöchentlich stattfindender Vorlesungs-Podcast, kommentierte Operationsvideos, Anamnesevideos, ein „Live-Patientenzimmer“ auf unserer eLearning-Präsenz sowie eine schriftliche Klausur, detaillierte Darstellungen und Evaluationsergebnisse hierzu wurden bereits publiziert [12].

### Erstellung der interaktiven Fälle

Die interaktiven Patientenfälle wurden von einem Team von 3 Ärzten verfasst, wobei 2 Weiterbildungsassistenten im 2. und 3. Weiterbildungsjahr als Autoren und einer als fachärztlicher Reviewer fungierten.

In einem ersten Schritt wurden 7 ophthalmologische Leitsymptome und -zeichen („red flags“) identifiziert, welche auf potenziell bedrohliche Verläufe hinweisend sein können und von Ärzten aller Fachrichtungen erkannt werden müssen [9]. Die Kriterien für die Auswahl waren akute Therapie- oder Abklärungsbedürftigkeit sowie potenziell visus- oder lebensbedrohliche Grunderkrankung.

Sieben Fälle zu den folgenden Themen wurden erstellt:

1. Schmerzen nach Trauma (bulbuspenetrierende Verletzung),
2. schmerzhafter Visusverlust (akutes Winkelblockglaukom),
3. schmerzhafter Visusverlust beim älteren Menschen (Riesenzellarteriitis),
4. schmerzloser Visusverlust (Zentralarterienverschluss),
5. neu aufgetretene Ptose (N.-oculomotorius-Läsion durch Aneurysma),
6. Rußregen/Blitze/Vorhangsehen (Amotio retinae),
7. rotes Auge (Endophthalmitis nach Kataraktoperation).

Für die einzelnen Leitsymptome bzw. Erkrankungen wurden Key-Features identifiziert (Tab. 1), also kritische Entscheidungen bzw. Abwägungen, welche notwendig sind, um einen entsprechenden Patienten sachgerecht zu versorgen [2, 10].

**Table 1:** 

Fall	Lernziele der Key-Features	Weitere wichtige, fallspezifische Lernziele
Schmerzen und Trauma (bulbuspenetrierende Verletzung)	Erkennung einer perforierenden Bulbusverletzung mit Fremdkörper durch Hammer-Meißel-Verletzung bei nur scheinbarem Bagatelltrauma Kontraindikation MRT bei möglichem metallischem Fremdkörper	Anamnese des Unfallmechanismus Diagnostische Möglichkeiten zur Abklärung des Verdachts eines intraokularen Fremdkörpers Spaltlampendiagnostik und Operation bei intraokularem Fremdkörper
Schmerzhafter Visusverlust (akutes Winkelblockglaukom)	Palpation als einfache diagnostische Option bei Kopfschmerz, Übelkeit und Sehverlust	Anamnese und weitere Symptome des akuten Winkelblockglaukoms Therapeutische Optionen
Rotes Auge (Endophthalmitis nach Kataraktoperation)	Erkennen von Schmerz, Rötung und Visusverlust nach Operation des Auges als Endophthalmitis Einschätzen der Dringlichkeit einer augenärztlichen Vorstellung	Differenzialdiagnose des roten Auges Therapeutische Optionen
Rußregen, Blitze, Gesichtsfeldeinschränkung (rhegmatogene Amotio retinae)	Erkennung der Befundkonstellation von wahrgenommenen Blitzen und „Vorhang“-Wahrnehmung Einschätzung der Dringlichkeit bei Makula anliegend vs. abgehoben	Differenzierte Anamnese bei „schwarzen Punkten“ Therapeutische Optionen
Schmerzloser Visusverlust (Zentralarterienverschluss)	Stellen der Verdachtsdiagnose von ischämischem Ereignis am Auge anhand des Leitsymptoms Ausschluss von arteriitischen Prozessen	Befunde und Differenzialdiagnostik mittels einfacher Untersuchungstechniken (Konfrontationsperimetrie, Pupillentestung) Notwendige kardiovaskuläre Abklärungen Lyse als therapeutische Option in Abwägung von Chancen und Risiken
Neue Ptose (N.-oculomotorius-Läsion durch Aneurysma)	Bedenken von Differenzialdiagnosen bei vermeintlich harmlosem Befund Diagnostik mit Pupillentestung und Motilität Veranlassung von bildgebenden Verfahren	Mögliche Ursachen, einseitiger vs. beidseitiger Befund Anisokorie, N.-oculomotorius-Parese, Horner-Syndrom
Schmerzhafter Visusverlust beim älteren Menschen (Riesenzellarteriitis)	Differenzierte Anamnese und Untersuchungen zur Risikostratifizierung einer Riesenzellarteriitis vs. nichtarteriitischen Prozessen Therapeutisches Management bei Verdacht auf Riesenzellarteriitis	Plötzlicher Sehverlust als Symptom von Erkrankungen des rheumatischen Formenkreises Unterschiedliche Ausprägung von Allgemeinsymptomatik bei Riesenzellarteriitis Komorbiditäten Therapiekonzept

**Figure 1:**
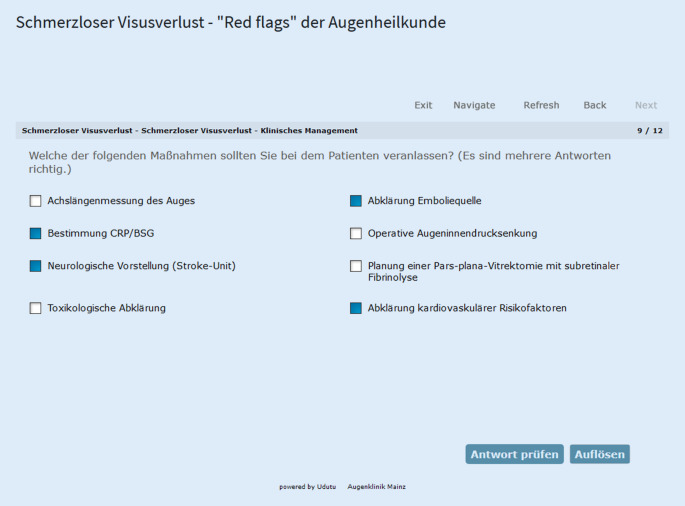

Auf der Basis der ausgewählten Key-Features wurde ein Skript erstellt, in dem die Patientenfälle mit Vignetten beschrieben wurden und zu ausgewählten Key-Features Fragen gestellt wurden. Fragen wurden in verschiedenen Formen eingebunden (Tab. 2; Abb. 1).

**Table 2:** 

Bezeichnung	Form	Beispiel
Multiple-Choice	Typ A (Einfachauswahl)	„Welche der genannten Diagnosen stellen Sie anhand Ihres Befundes?“
	Pick‑N (Mehrfachauswahl)	„Welche 3 der folgenden Differenzialdiagnosen stehen im Vordergrund?“
Freitext	Freitextreflexion mit Eingabebereich und Feedback-Button (mit richtiger Antwort und ausführlicher Begründung)	„Welche zielführenden Anamnesefragen stellen Sie der Patientin?“
Zuordnung („phrase matching“)	Frage mit bestimmter Anzahl an Textblöcken, denen dazugehörige Textblöcke richtig zugeordnet werden sollen	„Bitte ordnen Sie den genannten Medikamenten die korrekte Dosierung zu.“
„Hotspot“-Bildfrage	Frage mit Bild, in dem ein bestimmter, gesuchter Bereich markiert werden soll	„Bitte markieren Sie im Bild den maßgeblichsten Befund.“

**Figure 2:**
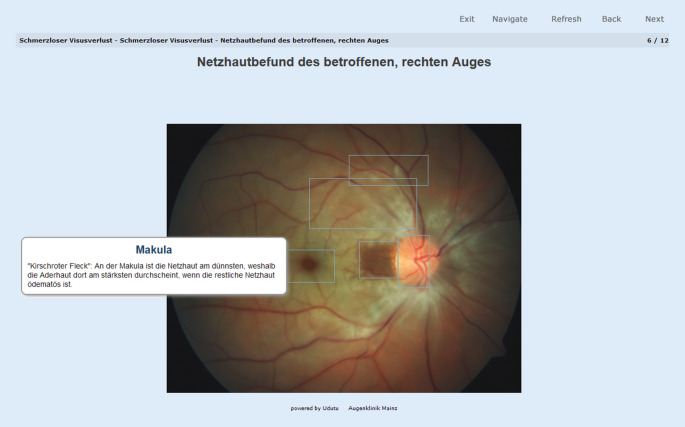

Die Fälle wurden mit Bildmaterial ergänzt, welches auch im Falle von mehreren oder diskreten Befunden mit „Hover-Funktion“ ausgestattet war, also an der Position des Mauszeigers ergänzende Informationen in einem separaten Textfenster angezeigt wurden (Abb. 2).

**Figure 3:**
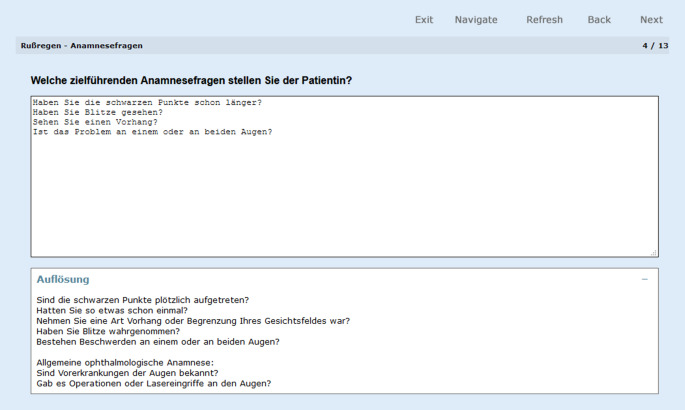

Freitextfragen erlaubten den Lernenden den Abgleich zwischen eigenem prozeduralem Denken und einer Musterlösung (Abb. 3).

Umgesetzt wurden die interaktiven Fälle mittels des Authoring-Tools (Online-Tool zur Erstellung von eLearning-Modulen) von udutu.com (Udutu Learning Systems Inc, Victoria, Kanada). Zum Zeitpunkt der Erstellung der Fälle konnte das Authoring-Tool-Angebot der Firma noch kostenlos genutzt werden, zum Stand 01/2021 steht ein Abonnementmodell zur Verfügung. Bei der Erstellung waren keine Programmierkenntnisse notwendig, es wurden lediglich einfache HTML-Codes eingebunden, um Freitextantworten zu ermöglichen.

Die interaktiven Fälle wurden in das Learning-Management-System (LMS) der Johannes Gutenberg-Universität Mainz eingebunden, auf welchem auch alle weiteren Lernmaterialien des Kurses zur Verfügung stehen (Moodle, Moodle Pty. Ltd., Perth, Australien). Die Fälle wurden konsekutiv im Laufe des Semesters freigeschaltet, wobei der Schwerpunkt hierbei im Juli lag (4/7 Fällen), damit die Studierenden bereits eine ausreichende theoretische Grundlage durch die Vorlesungen gewinnen konnten.

Neben der Authoring-Software und dem LMS waren weitere notwendige Materialien Abbildungen der Befunde sowie eine einfache Bildbearbeitungssoftware.

### Evaluation der interaktiven Fälle

Es nahmen 180 Studierende im 6. Fachsemester an der Evaluation nach Teilnahme an Vorlesung und Praktikum der Augenheilkunde der Poliklinik und Augenklinik der Universitätsmedizin Mainz im Sommersemester 2020 teil. Insgesamt 192 Studierende absolvierten das Praktikum und nahmen an der Klausur teil.

Die Studierenden wurden aufgefordert, nach Abschluss der Klausur einen anonymisierten Evaluationsbogen auszufüllen, welcher in Zusammenarbeit mit dem Zentrum für Qualitätssicherung und -entwicklung (ZQ) der Johannes Gutenberg-Universität Mainz gestaltet wurde. Die Teilnahme an der Evaluation war freiwillig und nicht mit Vorteilen oder einer Belohnung verbunden. Auf dem Evaluationsbogen wurden von den Studierenden zu den interaktiven Fällen sowie zur gesamten Veranstaltung Schulnoten vergeben und auf weiteren Likert-Skalen Aussagen zur Veranstaltung und der Einschätzung des individuellen Interesses an der Augenheilkunde bewertet. Weiterhin konnten Freitextantworten verfasst werden. Das ZQ wertete die Fragebögen anschließend standardisiert aus. Die Freitextantworten wurden durch einen Rater in die Kategorien „eher Lob“, „eher ausgewogen“ und „eher Kritik“ eingeteilt.

Außerdem wurden über das LMS Kurzevaluationen unmittelbar nach der Bearbeitung eines Falles durchgeführt, welche anonymisiert abgespeichert wurden. Wir werteten diese Daten zur Bewertung unserer Themenauswahl und möglichen auf einzelne interaktive Fälle bezogenen Fehlern oder Verbesserungsvorschlägen aus.

## Ergebnisse

Es konnten 164 Evaluationsbögen ausgewertet werden. Ausschlüsse erfolgten aufgrund von inkorrekt ausgefüllten Bögen oder Nicht-Nutzung des Lernangebotes. Für die jeweiligen Aussagen bzw. Fragen konnten zwischen *n* = 158 und *n* = 164 Fragebögen einbezogen werden. Die Freitextkommentare wurden zur internen Evaluation des Veranstaltungserfolges und der interaktiven Patientenfälle herangezogen und spiegelten die quantitativen Evaluationsergebnisse wider. Hierbei wurden insgesamt 26 Freitextkommentare ausgewertet. Von diesen waren 20 eher lobend, 4 eher kritisch und 2 eher gemischt. Inhaltlich wurde von den kritischen Kommentaren ausschließlich eine zu schlechte Vorbereitung der Fälle durch die Vorlesung benannt.

**Figure 4:**
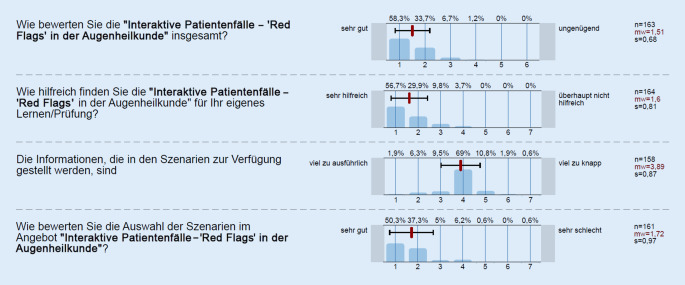

Übergreifend wurden die interaktiven Patientenfälle im Mittel mit einer Schulnote von 1,51 ± 0,68 (Mittelwert ± Standardabweichung; 1 = sehr gut, 6 = ungenügend) bewertet (*n* = 163). Die Verteilung und weitere Evaluationsaspekte sind in Abb. 4 dargestellt.

Für die Evaluationsergebnisse der einzelnen interaktiven Fälle wurden zwischen *n* = 135 und *n* = 105 Evaluationen in Moodle abgegeben. Die Tab. 3 stellt die Einzelbewertungen dar.

**Table 3:** 

Fälle nach Reihenfolge im Curriculum	*N*	Wie bewerten Sie den interaktiven Fall insgesamt? (Nach Schulnoten, 1 = sehr gut, 6 = ungenügend)	Wie bewerten Sie die Auswahl des Szenarios? (Likert-Skala 1 = sehr gut; 7 = sehr schlecht)	Waren die Informationen, die im Szenario zur Verfügung gestellt wurden, ausreichend? (Likert-Skala 1 = völlig ausreichend; 7 = viel zu gering)
Schmerzen und Trauma	135	1,62 ± 0,65	1,44 ± 0,58	1,79 ± 0,86
Schmerzhafter Visusverlust	121	1,63 ± 0,62	1,42 ± 0,56	1,82 ± 0,94
Rotes Auge	113	1,62 ± 0,68	1,5 ± 0,61	1,73 ± 0,94
Rußregen, Blitze, Vorhangsehen	106	1,79 ± 0,82	1,64 ± 0,68	2,02 ± 1,19
Schmerzloser Visusverlust	109	1,37 ± 0,57	1,36 ± 0,57	1,47 ± 0,7
Neue Ptose	106	1,4 ± 0,62	1,33 ± 0,51	1,5 ± 0,78
Schmerzhafter Visusverlust beim älteren Menschen	105	1,41 ± 0,55	1,52 ± 0,82	1,52 ± 0,87

## Diskussion

Patienten stellen sich mit ihren Symptomen und klinischen Zeichen, nicht mit ihrer Diagnose vor. Aus diesem Grund ist eine leitsymptomorientierte Didaktik wichtiger Bestandteil der studentischen Lehre, welcher in der Reform der medizinischen Studierendenausbildung im Rahmen des Masterplan 2020 und des Nationalen Kompetenzorientierten Lernzielkatalog Medizin (NKLM) im Kapitel 20 umfangreiche Berücksichtigung findet [7]. In der Augenheilkunde ist dieser Zugang aufgrund der fachspezifischen Symptome umso wichtiger, da nur einzelne bedrohliche ophthalmologische Erkrankungen mit ihren „red flags“ auch in anderen Fächern potenziell gelehrt werden, wie z. B. die Riesenzellarteriitis in der Neurologie.

Der NKLM fordert diese Hinwendung zu Leitsymptomen im Kapitel 20 auch von der Augenheilkunde, beispielsweise unter „20.12 Augenschmerzen“ oder „20.80 Rotes Auge“ als Konsultationsanlässe. Diese möchten wir in der im NKLM beschriebenen Kompetenzebene 2 „Handlungs- und Begründungswissen“ abbilden, um den Studierenden das Erreichen von Kompetenzebene 3 „Selbstständige Durchführung/Anwendung“ in praktischen Abschnitten ihrer weiteren Laufbahn zu ermöglichen.

Studierenden mittels interaktiver Fälle die bedrohlichen Leitsymptome der Augenheilkunde näherzubringen, wurde sehr gut akzeptiert und sowohl übergreifend, als auch in Bezug auf das eigene Lernen und die Szenarienauswahl überwiegend sehr gut bewertet.

Die Erstellung der Fälle gelang hierbei mit moderatem technischem Aufwand und geringen Investitionskosten. Wünschenswert wären allerdings weitere Frageformate gewesen, beispielsweise eine Long-Menu-Option hätte die Quizabschnitte deutlich aufgewertet [3]. Da jedoch aufgrund der verpflichtend digitalen Lehre im Rahmen der Corona-Pandemie keine Zeit für eine separate Programmierung und Implementierung entsprechender Features war, musste dies für die 1. Auflage der Fälle ausgespart werden [12].

Die ausgesprochen positive Rückmeldung der Studierenden zum Lernangebot und die umfangreichen und differenzierten Freitextevaluationen lassen eine rege Auseinandersetzung mit den Inhalten und dem Angebot vermuten. Überraschend für uns war, dass zwar in der Evaluation die Informationsmenge, welche zur Verfügung gestellt wurde, für die Lösung als genau richtig bewertet wurde, in den Freitextkommentaren jedoch darüber hinausgehend deutlich mehr Hintergründe zu den Fällen gewünscht wurden.

Die eher negativen Freitextkommentare der Studierenden merkten an, dass bei einer Generierung solcher oder ähnlicher neuer Angebote beachtet werden sollte, dass die neu geschaffenen Inhalte sich auch in anderen Lehrformaten, insbesondere den Vorlesungen, wiederfinden sollten. Die Verknüpfung von leitsymptomorientierten (z. B. interaktive Fälle) und organstrukturorientierten (z. B. Vorlesung) Lernangeboten ist daher eine Priorität für uns. Wir planen deshalb eine Überarbeitung unserer Vorlesungen hin zu mehr Symptombezug und Kompetenzorientierung, welche auch im NKLM gefordert ist. Ebenso möchten wir hiermit eine zusätzliche Ebene im Sinne eines repetitiven Lernansatzes bieten, um das Erreichen der prozeduralen Lernziele unserer interaktiven Fälle weiter zu fördern.

Gleichzeitig sehen wir die kritischen Kommentare auch als Bestätigung, dass wir mit den Fällen nicht nur Inhalte der Vorlesung wiederholt haben, sondern auch neue Inhalte, welche über eine theoretische Vorlesung hinausgehen, bieten konnten. Ein gewünschter Aspekt unseres digitalen Praktikums war eine solche Abbildung zusätzlicher Kompetenzen und Inhalte.

Ebenfalls wurde angemerkt, dass die Falsch- und Richtigantworten der Fälle ausführlich begründet werden sollten. Dies unterstreicht das große didaktische Potenzial, welches in der Einbindung formativer, also nicht für das Bestehen relevanter Prüfungs‑/Quizformate besteht [5].

Die ausgewählten Leitsymptome und klinischen Zeichen stellen unserer Ansicht nach lediglich die wichtigsten „red flags“ der Augenheilkunde dar. Sicherlich könnten zudem auch andere Inhalte abseits der Notfallmedizin vom dargestellten Format profitieren. Häufige Krankheitsbilder wie Diabetes oder altersabhängige Makuladegeneration mit ihren Therapiekonzepten und dem entsprechenden Management sollten auch nichtophthalmologisch betreuenden Ärzten geläufig sein. Weiterhin wurde je Leitsymptom nur eine der potenziellen Erkrankungen je Fall dargestellt. Wünschenswert wäre, zu jedem Leitsymptom möglichst mehrere, verschiedene Erkrankungen darzustellen.

Selbstverständlich ist das Format nicht auf die studentische Lehre beschränkt. Erfreulicherweise wird fallbasiertes Lernen auch für Weiterbildungsassistenten und Fachärzte immer digitaler [8]. Wir sehen in interaktiven Fallformaten großes Potenzial, Weiter- und Fortbildung zu bereichern. Sie erlauben nicht nur die Beschäftigung mit theoretischen Inhalten, sondern auch die Anwendung von prozeduralem Wissen. Einmalig geschaffene Angebote können so einer breiten Masse von Lernenden zugängig gemacht werden und könnten perspektivisch auch über die studentische Lehre hinaus ein wertvolles Lernformat darstellen.

## Ausblick

Das Konzept der eingerichteten interaktiven Fälle hat sich bewährt, weshalb wir diese weiter nutzen werden. Eine durch studentische Anregungen überarbeitete Version der interaktiven Fälle wird künftig als Pflichtbestandteil des Praktikums Augenheilkunde angeboten.

Für die Zukunft ist der Ausbau des Angebotes für weitere Leitsymptome und Erkrankungen vorgesehen.

## Fazit für die Praxis

- Interaktive Fälle zu den „red flags“ der Augenheilkunde sind eine wertvolle didaktische Möglichkeit, Studierenden die Notfälle und bedrohlichen Verläufe des Faches näherzubringen.
- Die Fälle wurden von den Studierenden ausgesprochen positiv aufgenommen und bekamen von ihnen eine hohe praktische Relevanz zugesprochen.
- Ein Ausbau mit zusätzlichen Fällen, welche über augenärztliche Notfälle hinausgehen, ist insbesondere für die ophthalmologische Weiter- und Fortbildung sinnvoll.
